# An introduction to scripting in Ruby for biologists

**DOI:** 10.1186/1471-2105-10-221

**Published:** 2009-07-16

**Authors:** Jan Aerts, Andy Law

**Affiliations:** 1 Genome Dynamics and Evolution Group, Wellcome Trust Sanger Institute, Wellcome Trust Genome Campus, Hinxton, CB10 1SA, UK; 2 Division of Genetics and Genomics, The Roslin Institute and Royal (Dick) School of Veterinary Studies, University of Edinburgh, Roslin, EH25 9PS, UK

## Abstract

The Ruby programming language has a lot to offer to any scientist with electronic data to process. Not only is the initial learning curve very shallow, but its reflection and meta-programming capabilities allow for the rapid creation of relatively complex applications while still keeping the code short and readable. This paper provides a gentle introduction to this scripting language for researchers without formal informatics training such as many wet-lab scientists. We hope this will provide such researchers an idea of how powerful a tool Ruby can be for their data management tasks and encourage them to learn more about it.

## Background

The past twenty years have seen an explosion in the quantity of data available to life scientists. Advances in DNA sequencing technology have resulted in ever cheaper and faster ways to sequence genomes, genotype individuals, essay gene and protein expression and otherwise generate raw data from biological samples. All fields of biology have been affected and the challenge facing the biologist now is how to most effectively analyze the vast amount of data available. Manual inspection has become impossible and scientists must use programmatic methods to filter raw data in order to interpret it or to provide the information required to generate or test new hypotheses.

Scripting has been defined as "... a way to automate repetitive tasks and handle large amounts of data" [1]. We believe that scripting will become an essential basic skill for any life-scientist, even for those who would consider themselves to be entirely wet lab-based.

There are many scripting languages available (including Perl, Python, Ruby and shell scripting languages), each of which has strengths and weaknesses as well as fanatical devotees and denigrators in almost equal measure. However, it should be noted that there is no single scripting language perfect for all use-cases. In addition, the interaction between the language syntax and constructs and the programmer's internal thought process is significant, such that what appears a perfectly logical choice and representation in one language to one programmer can appear obfuscated to another.

With all this in mind, in this short article we will introduce Ruby as a highly suitable scripting language for biologists to learn and use. We believe that Ruby is an excellent first step for people new to scripting. Its shallow learning curve combined with the consistency within the language make it easy to get started. At the same time, several properties of the language (e.g. the fact that built-in classes can be easily extended to fit different needs) make advanced scripting simple. Thus we also hope to provide exponents of other scripting/programming languages such as Perl, Python or Java some insights as to the capabilities of the Ruby language and encourage them to add an extra tool to their toolbox.

### Features of Ruby

Ruby is a programming (scripting) language devised by Yukihiro 'Matz' Matsumoto initially released for general use in 1995. It aims to achieve ease-of-use and, as Matsumoto claims in the foreword to the Programming Ruby book [2], "It allows you to concentrate on the creative side of programming, with less stress". It provides a lot of functionality on which the programmer can build without having to worry about the underlying mechanics. Like many other programming languages, it borrows heavily from pre-existing languages (*i.c*. Perl, Smalltalk and Lisp).

#### Interpreted language

In order for a computer to execute a set of instructions, those instructions need to be converted into a form that the machine processor can understand. The process of conversion from high-level, human-readable code into machine-executable instructions can be done either on the fly as and when needed, or as an explicit step during development. The distinction between these two approaches separates languages into 'compiled' (i.e. convert to machine executable as a separate step) and 'interpreted' (i.e. convert as required) subsets. Compiled languages offer advantages in terms of speed of execution of the finished program whereas interpreted languages often have the edge when it comes to overall speed of development. The latter is partly due to elimination of compilation time, but also to the fact that scripting languages typically work at a more abstract or higher level than compiled languages, which means that a single statement on average does more work [3], leading to shorter programs.

#### Object-orientation

One of the primary features of Ruby is that it is a strict Object-Oriented (OO) language. Everything in Ruby is an object with defined attributes that can be manipulated using associated methods. Objects are instances of classes, which describe the object concept. For example an object "Tom" can be an instance of the Person class. In this respect it is similar to Smalltalk and Python and less so to Perl which, although Perl can be used as an OO language, is more often to be found utilized as procedural code. This is an important point as it allows the Ruby language to provide a lot of built-in functionality in an extensible way.

To illustrate the difference Listings 1 and 2 contain some equivalent code fragments in Perl and in Ruby which read a series of lines from a file (e.g. called 'my file.txt') and print them out with each line reversed. Both code fragments will produce identical output given the same input file.

In the Perl version (Listing 1), the script opens the file, then pulls lines from it one by one. For each line, it removes trailing line-feed characters (chomp()), splits it into individual characters, reverses the order of those characters and then re-assembles the string which it prints out. In Perl, a string is simply a concatenation of characters. Therefore, the programmer has to explicitly write code split each string into an array of characters, reverse that array and then join the characters back up into a new string.

**Listing 1 **A Perl script to reverse a series of lines from a file

open INFILE, "my_file.txt";

while (defined ($line = <INFILE>)) {

   chomp($line);

   @letters = split(//, $line);

   @reverse_letters = reverse(@letters);

   $reverse_string = join("", @reverse_letters);

   print $reverse_string, "\n";

}

In the Ruby version (Listing 2), the script creates a file object using a built-in routine (File.open("my_file.txt")). Because files are objects in Ruby, they 'know' how to do certain things like return a list of all the lines that the file contains (each). This list can be passed to a block (do ... end) that loops over the list putting each line in turn into a variable placeholder called line. Since each line is a string object rather than just a concatenation of characters, it comes packed with added functionality: it can be told to remove trailing line-feed characters from itself (line.chomp) and then to reverse itself as well.

**Listing 2 **A Ruby script to reverse a series of lines from a file

File.open("my_file.txt").each do |line|

   puts line.chomp.reverse

end

Even though the distinction between these two approaches might not be entirely obvious to the uninitiated, it is profound. A program written in a procedural way (such as the Perl example) provides a list of tasks that have to be performed by the computer. An object-oriented program, however, can be seen as a collection of objects that interact with each other by sending messages. Each object is an instance of a general class that describes a concept (for example the String class) and carries its own operators (e.g. chomp and reverse). In the Perl version, it is the script that has to handle the file, read lines (strings) from that file one by one and then perform explicit actions on those strings. The programmer needs to know that each line can be converted into an array of characters and that the array can be reversed and then converted back into a string. In the Ruby version, however, the script acts at a higher level. It creates objects representing the file and the lines and then tells those objects to produce the desired effect. It is the objects that are responsible for the mechanics of implementation of that behaviour. The script does not care about the specifics of the implementation; it is only concerned with the end result.

The example in Perl code can be made shorter or implemented in an object-oriented way by loading additional Perl libraries. However, we believe that people new to programming are more likely to start working with unmodified (so-called "vanilla") installations of a programming language.

#### Class definition

Listing 3 shows a simple Ruby script illustrating some of the basic concepts. The line numbering is added here for explanatory purposes and is not part of the program (and should therefore not be in the script file). Anything preceded with a hash symbol (#) is a comment inserted purely for better human comprehension of the code and will be skipped by the interpreter.

**Listing 3 **A simple Ruby object to demonstrate some basic principles

1 # Let's start with a class definition

2 class Person

3   attr_accessor :name

4   def identify

5      puts "hello - my name is " + @name

6   end

7 end

8

9 # and then use that class to do something

10 person_one = Person.new

11 person_one = 'Tom'

12 person_two = Person.new

13 person_two.name = 'Nathalie'

14 person_one.identify

15 person_two.identify

This short program generates a Person class, representing the concept of a person. Note that class names must be capitalised. We first describe the class and what it should do, defining a Person as being an object with a (settable and retrievable) name that can report its name when asked to identify itself. The attr_accessor :name code on line 3 makes it possible to access the name property of a person as on lines 11 and 13.

Line 10 creates an instance (or object) of the class Person and puts it in a variable called person_one (variable names begin with lowercase letters). Line 11 sets the name of this particular Person (person_one) to be 'Tom'. Lines 12 and 13 do the same for a second instance of Person (person_two, called 'Nathalie'). Lines 14 and 15 tell each Person instance in turn to report their name. Because the Person's @name attribute is a so-called 'instance' variable, each instance of Person has its own name. All person objects are independent of each other but share the same behaviour.

This is admittedly a trivial example, but imagine a more complex example where each Person has @father and @mother attributes. With a single object class definition, it becomes possible to implement and explore an entire family tree.

Classes that describe concepts within a well-defined domain can be functionally grouped into so-called modules. A relevant example here is the Bio module provided by the BioRuby project [4]. BioRuby is an effort by a global group of bioinformaticians (led from Japan) to create such module in the domain of biological research. This module describes the concepts of a DNA or protein sequence with their different representations (e.g. EMBL, GenBank, Fasta), an alignment, the results from a similarity search, pathways, references and other biological/bioinformatics concepts. For example, the Bio::Sequence class holds all functionality associated with a biological sequence. Any object of that class can generate the reverse complement of itself (e.g. puts Bio::Sequence.new('AATGCC').complement). This module does not yet provide all functionality that is available in the sister-project BioPerl for the Perl language, but can already be of much value for writing biology-related scripts. See the BioRuby website for more details of the functionality of the classes that this module provides.

#### Blocks

One powerful feature of Ruby is its use of blocks. Blocks are nameless functions that can be passed to other functions easily and do not have to be defined before being used. Take for example a collection of people. The approach to traverse this collection often used in scripting languages is a for-loop as shown in section A of Listing 4. In contrast, Ruby scripts typically use the each iterator as exemplified in section B. As the name implies, iterators are methods that iterate over a collection and perform a task on each element of that collection.

The each method takes a block as its argument (either delineated by "do..end" or by curly braces) and substitutes the variable person within the block with each consecutive value in the people collection. Although each is a method defined in the Array class, iterators can be defined on other classes as well. A simple example is shown in Listing 4 section C. This code will print "Hello world" five times. The times method is defined in the Integer class.

Another common task to be performed on collections is to select those elements that comply to a given criterion. To create a list containing only those people younger than 20 years, for example, one could write the code in section D of Listing 4. Blocks however make it possible to make an abstraction of this looping. The select method as shown in section E of Listing 4 has a single block argument which defines the criteria for selection.

Finally, the strength of blocks can be illustrated with the sort_by method which takes a block that defines the rules for sorting. Section F of Listing 4 shows a very simple example where the people are sorted only by age, but more complicated sorting algorithms can easily be implemented by changing the code within the block.

Although it is possible and even common to create custom loop abstractions (e.g. a function that would traverse a tree depth-first) and create methods that take a block as argument, this functionality is typically not used by people new to programming and therefore exceeds the scope of this article.

**Listing 4 **Using blocks in Ruby

1 # A. Using a for-loop

2 for person in people

3   puts person.name

4 end

5

6 # B. Using the each iterator

7 people.each do |person|

8   puts person.name

9 end

10

11 # C. Doing something 5 times

12 5.times do

13   puts "Hello world"

14 end

15

16 # D. Making a subselection for a collection

17 young_people = Array.new

18 for person in people

19   if person.age < 20

20      young_people.push(person)

21   end

22 end

23

24 # E. Making a subselection for a collection using a block

25 young_people = people.select{|person| person.age < 20}

26

27 # F. Sorting people

28 people_sorted_by_age = people.sort_by{|person| person.age}

#### Reflection

Ruby classes and objects have the ability to reflect on their own states and functionality. For example, every object implements a methods function that looks inside the object's own definition and returns a list of methods that the object implements. Consequently, because everything is an object and every object can be queried for its methods it is very simple to determine how to accomplish tasks. For example, asking a String object for its list of methods results in a list of 145 functions, including reverse, capitalize, chomp (as used in the example above), swapcase and length.

#### Meta-programming

Because Ruby classes and objects can reflect on themselves, meta-programming is easy in Ruby. Meta-programming refers to either writing code that writes code, or to changing code at runtime, i.e. while the program is running. A good example of this is demonstrated in Listing 3. The attr_accessor :name code on line 3 is actually translated automatically into methods that allow any code using a Person object to access (get or set) the object's name field. If we had wanted only to allow getting or setting of the name field, we could have used the alternatives attr_reader or attr_writer instead.

Meta-programming often starts with providing a method_missing method in a class. This acts as a catch-all for methods that do not exist for that class. Listing 5 provides a trivial example. Using this addition to the standard Integer class, it is possible to use methods such as multiply_3 or add_9, even though they are never explicitly defined. When calling 5.multiply_3, for example, ruby will notice that that method does not exist and diverts it to method_missing. This approach is the basis for much of the ActiveRecord module (see below) that provides a find_by_ method for any column in a database table without having to explicitly define each.

**Listing 5 **A simple meta-programming example

1 class Integer

2   def method_missing(method_name)

3      command, number = method_name.to_s.split(/_/)

4      if command == 'multiply'

5         return self * number.to_i

6      elsif command == 'add'

7         return self + number.to_i

9   end

10 end

## Results

To demonstrate the way Ruby can be used in bioinformatics, we will briefly present three simple examples.

### Wrapping repetitive commands to run in batch

The authors recently worked on a project in which they needed to run an analysis program (spidey, [5]) that takes two inputs – the first a file containing a number of transcript sequences and the second a file with a single genomic sequence. The general format of the command to be run was:

spidey -m transcript_sequences.fa -i genomic_sequence.fa >> output.txt

where transcript_sequences.fa is a FASTA-formatted file containing one or more transcript sequences; genomic_sequence.fa is a FASTA file with a single genomic DNA sequence. The results of the analysis are appended to a file called output.txt.

Unfortunately, the genome in question was not completely assembled beyond the scaffold stage and was therefore represented by thousands of separate sequence files. Running the command manually against each of those genomic fragments in turn was not a practical proposition. Instead, we used Ruby to deal with the repetition for us.

In Listing 2 we used a Ruby built-in class called File to open a file on disk and return each line that it contained. Here we used a similar built-in class called Dir to return a list of all the filenames within a directory (Listing 6). We were then able to use the Ruby system command to run the spidey program, inserting each filename in turn into the relevant position.

**Listing 6 **Wrapping repetitive commands in Ruby

Dir.foreach(".") do |filename|

   system("spidey -m transcript_sequences.fa -i " + filename + " >> output.txt")

end

### Extracting information from a text file

Researchers are often presented with the challenge of selecting a subset of lines or columns from a datafile, i.e. filtering data. This can be done with Microsoft Excel or standard linux shell commands, but a script becomes necessary for more complicated problems.

Here we will use a list of features described in a GFF file (General Feature Format, [6]) that resembles Table 1 and identify those features that overlap with a given region. An example file containing a small quantity of GFF-formatted data is provided as Additional File 1. To find out which of the features (i.e. genes) are contained within a target range of 300,000 to 450,000 basepairs, we can use the script in Listing 7.

**Table 1:** Example data in GFF format

chr1	Ensembl	gene	40344	40376	.	+	.	Accession "ENSG00000177757"
chr1	Ensembl	gene	134390	134439	.	+	.	Accession "ENSG00000177750"
chr1	Ensembl	gene	394524	394570	.	+	.	Accession "ENSG00000177741"
chr1	Ensembl	gene	412258	412298	.	+	.	Accession "ENSG00000187583"
chr1	Ensembl	gene	552024	552064	.	+	.	Accession "ENSG00000187642"

**Listing 7 **Finding features in a range

1 class Range

2   def contained_by?(other_range)

3      if self.begin > other_range.begin and self.end < other_range.end

4         return true

5      else

6         return false

7      end

8   end

9 end

10

11 class GffRecord

12   attr_accessor :seq_id, :source, :type, :start, :stop, :score, :strand, :phase, :attributes

13   def initialize(gff_line)

14      @seq_id, @source, @type, @start, @stop, @score, @strand, @phase, @attributes = gff_line.split(/\t/)

15   end

16

17   def range

18      return Range.new(@start.to_i, @stop.to_i)

19   end

20 end

21

22 target_range = Range.new(300000,450000)

23 File.open('additional_file_1.txt').each do |line|

24   line.chomp!

25   gff_record = GffRecord.new(line)

26   if gff_record.range.contained_by?(target_range)

27      puts gff_record.attributes

28   end

29 end

In lines 23 to 29 of the script, each line from the datafile is read and the feature it describes is reported if it falls completely within the target range we set at line 22.

In more detail, line 23 reads the file and passes each line one by one to the block defined between lines 24 and 29. Line 24 removes the newline at the end of the input line and the line is passed as the argument to create a new GffRecord object. Next, the range contained in that GFF record is tested to lie within a target range. If so, the 'attributes' part of the GFF record is printed to the screen.

This example shows how, typically, the part of the script containing the business logic is quite short (i.e. from line 22 to 29). It is the class definitions that precede it that make this possible. In line 1 to 9 a new method is added to the built-in Range class (which is then used on line 26). This class is used to represent intervals, i.e. sets of values with a start and an end (e.g. '1 to 5'). Lines 11 to 20 define our new GffRecord class. The initialize method handles the creation of a new object, which should be given a single line of a GFF file (see line 25). This line is then split on tabs and each component is put in its particular instance variable (line 14). In addition to the initialize method, we also define a range method (lines 17 to 19). This method returns a Range object using the object start and stop positions as boundaries. Because we return a Range object, and because we have already extended the Range class (and hence all Range objects regardless of their origin), we can use the contained_by? method to determine if this is a record in which we are interested.

The above example demonstrates a further useful property of the language: class definitions are open. This means that it is possible to alter existing and even built-in class definitions at runtime to add or change behaviour. The standard Range class does not have a contained_by? method. Here we just added this functionality on the fly by defining it between lines 1 and 9, retaining the full existing functionality of the Range class but extending it for our needs.

Typically, people writing scripts will start to reuse the same class definitions (e.g. for a GffRecord) which then can be put together in a central library. As a result, many scripts will end up containing only the business logic (i.e. lines 22 to 29 above) and the custom class definitions as presented here can be omitted.

### Accessing data in a database

Most bioinformaticians will agree that a database is often the best tool for storing data. It is therefore an important consideration when choosing a scripting language that it provide an easy way of interacting with databases. Database interaction in Ruby is typically performed via the ActiveRecord module.

ActiveRecord is a Ruby module that allows easy interfacing with the tables in a database. Using meta-programming as described above, it connects database tables to Ruby classes, linking each record in a table to a single object (i.e. instance) of the corresponding class. This provides the scripter with a double layer of abstraction – not only hiding away the specific database engine implementation details (c.f. the Perl DBI or Java JDBC interfaces) but also completely removing the need to write SQL (Structured Query Language) if the database owner agrees to follow a few simple naming conventions. This is a theme that recurs throughout Ruby, particularly in the ActiveRecord framework: convention over configuration. It is possible to configure Ruby to handle scenarios which don't map to the convention but to do so is much harder than to just 'go with the flow' of convention. This of course leaves the bioinformatician with the choice: either follow strict rules on how to organize your data, or add a lot of configurations in your scripts. It is our opinion that if possible general conventions should be used to simplify Ruby scripts.

If a database schema follows the documented conventions, ActiveRecord can access the data automatically through a process called Object/Relationship Mapping (ORM). The conventions that are relevant here are: (1) table names have to be plural, (2) the primary key must be called id, and (3) foreign keys to other tables should consist of the singular form of that table name followed by _id.

Listing 8 provides an example of how to interact with an existing database. Suppose the data from the GFF file in the previous example was stored in a MySQL database with three tables, called genes, mappings and transcripts. Additional File 2 contains the SQL commands to create a minimal mySQL relational database with the same data as in Additional File 1. The genes table contains the columns id, name and accession; the mappings table contains the columns id, gene id (foreign key to the genes table), chromosome, start and stop; the transcripts table has the columns id, gene id (foreign key to the genes table) and accession. A simple SQL script suitable for loading example data that can be used with the code in Listing 8 is provided as an additional file. The script illustrates the use of ActiveRecord (and again reuses our Range class extension from the previous example).

Output of the script using the data in Additional File 2 will be:

Q96BN7_HUMAN is in the range 300,000 – 450,000

PLEKHN1 is in the range 300,000 – 450,000

Transcript for ENSG00000187583: ENST00000379409

Transcript for ENSG00000187583: ENST00000379410

Transcript for ENSG00000187583: ENST00000379407

Many things are happening automatically. First of all, any class that inherits from ActiveRecord::Base (< ActiveRecord::Base) is automatically linked to the table that has the lowercase plural version of the class name. Secondly, using has_many, has_one and belongs_to creates methods for every gene to get its mapping and all its transcripts. To get the mapping for a gene (my_gene), you call my_gene.mapping; to get the transcripts for a gene, you call my_gene.transcripts. The latter returns a list of transcript objects. In many cases, this list will only contain one element, but sometimes (e.g. for PLEKHN1) multiple transcripts will be returned. Similar to line.chomp.reverse in Listing 2, line 38 shows how different methods can be concatenated: g.mapping.start calls the start method on g.mapping, which itself is a call of the mapping method on the object g. Tables can be queried by any column, by appending the column name to find_by_, as exemplified in Gene.find_by_accession (task B in the script).

**Listing 8 **Extracting data from database

1 require 'activerecord'

2

3 class Range

4   def contained_by?(other_range)

5      if self.begin > other_range.begin and self.end < other_range.end

6         return true

7      else

8         return false

9      end

10   end

11 end

12

13 # Establish the connection to the database

14 # and create the classes.

15 ActiveRecord::Base.establish_connection(

16   :adapter => 'mysql',

17   :database => 'my_database'

18 )

19

20 class Gene < ActiveRecord::Base

21   has_one :mapping

22   has_many :transcripts

23 end

24

25 class Mapping < ActiveRecord::Base

26   belongs_to :gene

27 end

28

29 class Transcript < ActiveRecord::Base

30   belongs_to :gene

31 end

32

33 # And then use the classes to do some tasks:

34 # A. Find all genes in a certain region

35 target_range = Range.new(300000,450000)

36 all_genes = Gene.find(:all)

37 all_genes.each do |g|

38   gene_range = Range.new(g.mapping.start,g.mapping.stop)

39   if gene_range.contained_by?(target_range)

40      puts g.name + " is in the range 300,000 – 450,000"

41 end

42 end

43

44 # B. Find the transcripts for ENSG00000187583

45 my_gene = Gene.find_by_accession('ENSG00000187583')

46 my_gene.transcripts.each do |t|

47   puts "Transcript for ENSG00000187583: " + t.accession

48 end

## Conclusion

We hope that this gentle introduction to the Ruby programming language provides a framework for biologists to start investigating scripting in general. Given the large amounts of data that are often generated in research, such as sequences and expression data, scripting might become a basic tool for researchers in general and not only bioinformaticians.

We believe the Ruby language is a good stepping stone for people not familiar with programming due to its shallow learning curve, its conciseness, the way iterations are handled and the well-established modules for handling data such as ActiveRecord.

## Further information

There is a plethora of information sources available to assist the reader first in learning Ruby and subsequently in using it in (biological) research. Many websites provide documentation, tutorials and advice for Ruby programmers of all levels from novice to expert. These include the official Ruby website [7], the 'poignant guide to Ruby' [8], and a website that allows the user to try out Ruby from within a browser [9]. In addition, a large number of books are available describing the language, including Programming Ruby: The Pragmatic Programmers' Guide [2], the Ruby Cookbook [10] and Learning Ruby [11].

## Authors' contributions

JA provided the original idea and the impetus to write the paper. JA and AL contributed equally to the writing of the document. Both authors read and approved the final manuscript.

## Supplementary Material

Additional file 1 **Example data in GFF format**. A short example file containing GFF-formatted data for use with Listing 7. Click here for file

Additional file 2 **SQL loading script**. A SQL script to load data into a database suitable for use with Listing 8. Click here for file
